# Dissemination of knowledge from Cochrane systematic reviews in public health: Cross-sectional study

**DOI:** 10.1093/eurpub/ckac131.565

**Published:** 2022-10-25

**Authors:** SM Helmer, L Mergenthal, K De Santis, K Matthias

**Affiliations:** 1 Human and Health Sciences, University Bremen, Bremen, Germany; 2 Cochrane Public Health Europe, Bremen, Germany; 3 Department of Prevention and Evaluation, Leibniz BIPS, Bremen, Germany; 4 Faculty of Electrical Engineering, University of Applied Science Stralsund, Stralsund, Germany

## Abstract

**Background:**

Appropriate dissemination of public health evidence is of high importance to ensure that relevant knowledge reaches potential stakeholders and relevant population groups. A wide distrust towards science and its findings indicates that communication thereof remains below its potential. Cochrane Public Health (CPH) provides an important source of high-quality scientific evidence. This study aimed to identify (1) dissemination strategies and (2) possible stakeholders of Cochrane Public Health reviews.

**Methods:**

This is a cross-sectional, meta-research study. All 68 records (reviews or protocols) listed on the CPH website https://ph.cochrane.org/cph-reviews-and-topics up to 08.03.2022 were included. Record characteristics, dissemination strategy information and potential stakeholder details were coded by one author and 10% of records were checked by another author. Data were descriptively analysed.

**Results:**

53 reviews (46 systematic reviews, 6 rapid reviews, 1 scoping review) and 15 review protocols were included. The 53 reviews were published between 2010-2022 and included 1-153 primary studies. All reviews had an open-access plain language summary (PLS) in English with translations in 3-13 other languages. Although 16 of 53 reviews and 4 of 15 protocols reported any involvement in the review process of an advisory group, only 3 of 68 records included a dissemination plan aiming to inform non-academic audiences or policy.

**Conclusions:**

All identified records can be considered as relevant to a wide range of stakeholders and population groups. However, CPH reviews or protocols rarely report their dissemination strategies. It is unclear what dissemination strategies are used after CPH reviews are published. High relevance of CPH evidence for non-academic stakeholders and the general population highlights the need for adequate knowledge translation beyond academia.

**Key messages:**

• Dissemination plans and implementation is rarely reported in CPH reviews.

• Evidence from CPH reviews is relevant for a multitude of stakeholders.

